# Global research trends in the subjective well-being of older adults from 2002 to 2021: A bibliometric analysis

**DOI:** 10.3389/fpsyg.2022.972515

**Published:** 2022-09-09

**Authors:** Derong Huang, Jian Wang, Huiling Fang, Xuehan Wang, Yujie Zhang, Shuo Cao

**Affiliations:** ^1^Centre for Health Management and Policy Research, School of Public Health, Cheeloo College of Medicine, Shandong University, Jinan, China; ^2^NHC KeyLab of Health Economics and Policy Research, Shandong University, Jinan, China; ^3^School of Public Administration, Central South University, Changsha, Hunan, China

**Keywords:** subjective well-being, older adults, CiteSpace, bibliometric analysis, research trends

## Abstract

**Purpose:**

This study aimed to explore current developments and trends in the field of subjective well-being (SWB) of older adults at a macro level and identify research hotspots.

**Methods:**

We included reviews and articles on the SWB of older adults in the Web of Science Core Collection published from 2002 to 2021. We used CiteSpace to draw a knowledge map of the authors, institutions, countries, references, and keywords for visual analysis and used Microsoft Excel tables to count basic information details.

**Results:**

A total of 354 papers were included, and the number of papers published over the past two decades showed a pattern of growth. The core force of publications was primarily attributed to studies conducted in Europe, North America, Asia, and Oceania, which have relatively major issues of aging and good economic strength. However, links between states, institutions, and authors were relatively weak. Cluster analysis showed that the research field could be divided into eight topics: the application of social psychology in the study of the SWB of older adults, aging in older adults, health condition of older adults, achieving successful aging, interventions for SWB, age differences in SWB research, an economic perspective of SWB research and social support for older adults. Current research frontiers are socioeconomic status, community, intervention, participation, adjustment, validation, and personality.

**Conclusion:**

The results of the present study provided a comprehensive picture in the research field of SWB of older adults. It showed that the mechanism, especially the bidirectional effect, between the SWB of older adults and its influencing factors is still worthy of further exploration. More research on evidence-based and intervention strategies should be conducted in the future.

## Introduction

Using gross domestic product as the main indicator of social progress has been criticized in the past. Since the beginning of the century, well-being has been gradually receiving increasing attention from governments in the field of policy making (Austin, 2016). It is predicted that the proportion of the older adult population globally will rise from 12.8% in 2017 to 20% in 2050 (United Nations [UN], 2017). Population aging has repercussions for social economy, welfare, pension and many other aspects, and has become one of the main challenges faced by countries around the world. Population aging is the product of social and economic development and demonstrates the remarkable achievements of national economic development. Therefore, with the increase in the older adult population, improving the well-being of older adults has become an important research area. However, socio-economic development does not consistently improve well-being. Indeed, studies on the association between income and well-being are inconsistent. Most studies have confirmed that income is positively correlated with well-being (Howell and Howell, 2008), although some have supported the notion of diminishing marginal utility of income (Layard et al., 2008), suggesting that the relationship between income and well-being weakens with increasing income. Whether there is a saturation point between income and well-being remains controversial (Jebb et al., 2018). Currently, it appears that economic development only meets the basic material needs of the public and does not continuously improve well-being. Moreover, older adults face heavier burdens of disease and negative emotions and are at a higher risk of mental health deterioration (Bunce et al., 2008; de Mendonca Lima and Ivbijaro, 2013). Creating an environment and living conditions that support well-being can improve mental health (World Health Organization [WHO], 2017). Thus, improving the well-being of older adults is not only an important topic but also a complex research area.

Presently, there are three main research orientations of well-being: subjective well-being, psychological well-being and social well-being. Subjective well-being mainly refers to an individual’s overall evaluation of life quality at a particular stage according to the standards set by oneself (Diener et al., 1999). Subjective well-being is generally considered to include two components, an affective (emotional) component balanced between positive affect and negative affect, and a cognitive component based on life satisfaction (Diener, 1984). Psychological well-being is more concerned with personal growth and development, mainly including aspects such as self-acceptance and life goals (Ryff, 1989). Social well-being pays more attention to the optimal functioning of individuals in social life, and it includes five dimensions: social acceptance, social actualization, social contribution, social coherence, and social integration (Keyes, 1998). The research content of the three well-being types is related but empirically different (Keyes, 2002; Robitschek and Keyes, 2009). Of these, subjective well-being is the mainstream research orientation, and is often used as an essential indicator in large social surveys. Therefore, we have only explored subjective well-being in this paper.

Although several reviews on SWB have been published, traditional reviews are generally highly subjective and tend to have some limitations, such as the inability to identify some potential research trends, and the most important point is the inability to objectively show readers the relatively comprehensive picture of the research field. Furthermore, there are relatively few reviews on SWB of the older adult population, and the literature has not been analyzed using a bibliometric approach. Different from traditional review, bibliometric review refers to the use of bibliometric methods combined with systematic mapping tools to visually display the mutual influence of literature, institutions, scholars, etc., in a certain field for researchers. It can reveal the development of this field and help researchers to discover potential research trends and hotspots. Thus, in this study, we used CiteSpace to visually analyze the international research field of SWB in older adults over the past 20 years. Specifically, the research questions were as follows:

1) Which journals or disciplines are most influential in this field?
2) Which institutions, authors, countries, and literature contributed the most to this field?
3) What are the main research topics in this field?
4) What are the research trends and changes in this field?

## Methods

### Data collection

The purpose of this study was not to offer a highly comprehensive and systematic overview but to observe the dynamics of the field via the core literature and capture vital research frontiers. We used the Web of Science Core Collection as the literature source database. The search formula has been fine-tuned as follows: TS = “subject* well-being” AND TI = (“old* person” OR “old* people” OR “old* population” OR “old* man” OR “old* woman” OR “old* adult” OR “late* life” OR “very old” OR “fourth age” OR “oldest-old” OR retirement OR elder* OR aging OR geriatric* OR senior*), and literature type was restricted to reviews or articles published from January 1, 2002, to December 31, 2021, in any language. A total of 562 articles were retrieved. To improve the quality of literature data, we screened the literature according to the following exclusion criteria: (1) the research topic was not about the SWB of older adults; (2) the main research object was not the older adult population or involved other groups; (3) non-research literature. Finally, we obtained 354 papers, for which all records and references were exported in plain text format. Data were cleaned, and missing time entries in records were added. In addition, publication time was considered the actual publication time, where the network release time was adjusted (e.g., if the online publication date of an article was 2022, this was adjusted to the actual publication date of 2021).

### Data analysis

We imported the literature data into CiteSpace and set the time span as 2002–2021, the time slice as always 1 (1 year as a unit of observation), and the threshold as mainly TopN = 100 (select top 100 levels of most cited or occurred items from each slice). Nodes such as “Institution,” “Author,” “Reference,” “Country,” and “Keywords” were selected to generate knowledge maps of the collaborative network of the institution, author, country, co-citation cluster, the co-occurrence of keywords, and the emergence of keywords to conduct a visual analysis of related fields. In addition, we used the Microsoft Excel software to sort the relevant data generated by CiteSpace and generate bar charts and tables to further illustrate the data characteristics.

CiteSpace is a bibliometric software based on a Java program invented by Mr. Chen Chaomei. It can transform the social network relationship of the key elements of an article into visual knowledge maps. It has been used widely in various disciplines in recent years, and numerous CiteSpace reviews have been published (Chen et al., 2020; Song et al., 2022). The knowledge map is composed primarily of nodes and lines. A node represents a key element (e.g., an author or keyword), and the size of the node represents the frequency of occurrence of the key element. The thickness of the line between nodes represents the strength of the relationship between nodes. The betweenness centrality calculates the number of times a node appears on the shortest path between other nodes (Freeman, 1977). A betweenness centrality ≥ 0.1 is considered to indicate high betweenness centrality. Nodes with high betweenness centrality usually indicate turning points in the network. The upper left corner of the knowledge map shows the overall digital characteristics, such as the number of nodes and network density, as well as the setting of thresholds. Nodes or other changes adjust the map, and scientific and reasonable maps according to specific expectations can usually be drawn. In the clustering analysis, modularity (Q value) is used to measure the ability of a network to divide modules or clusters, Q value > 0.3 indicates a significant internal structure of the cluster. Mean silhouette (S value) is used to explain and verify cluster internal consistency (Shibata et al., 2008), S value > 0.5 indicates reasonable and reliable clustering results. Therefore, Q and S values should be used to measure the scientific nature of the generation of the knowledge map.

## Results

### Basic statistical analysis

**FIGURE 1:**
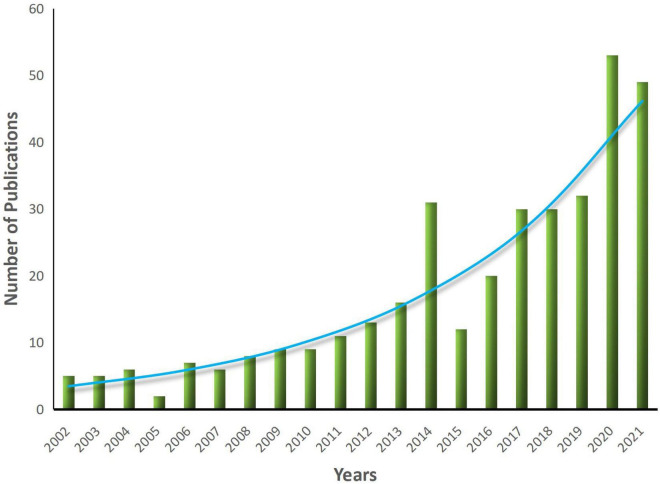
Statistics and trends of annual publications from 2002 to 2021.

Although SWB appeared early, relevant papers on the SWB of older adults were limited at the beginning of the century (Figure 1) and remained scarce from 2002 to 2007. After 2007, studies on SWB began to increase and first peaked in 2014; however, there was a slight decline in 2015. Nevertheless, the increasing trend continued and the number of studies peaked again in 2020. Overall, relatively little attention was paid to older adults in the research field of SWB during the early 21th century. However, in the past decade, relevant research has shown rapid growth overall.

### Journal analysis

A total of 354 articles were retrieved from 183 journals. Table 1 shows the top 10 journals. *Aging and Mental Health* published the most articles, and *Journals of Gerontology Series B-Psychological Sciences and Social Sciences, International Journal of Environmental Research and Public Health*, and other journals also published a high number of articles, which mainly involved gerontology, psychology, public health, sociology, and other disciplines. Among these is the *Journal of Happiness Studies*, which is a journal that has a leading research theme of happiness. Most journals had an impact factor of 3–4 for 2021, and the *Gerontologist* was the only journal that exceeded an impact factor of 5.

**TABLE 1:** Summary of the publications of the top 10 journals.

Journal	*N* (%)	Country	Impact factor (2021)
Aging and Mental Health	17 (4.80)	England	3.514
Journals of Gerontology Series B-Psychological Sciences and Social Sciences	16 (4.52)	United States	4.942
International Journal of Environmental Research and Public Health	14 (3.95)	Switzerland	4.614
Journal of Happiness Studies	14 (3.95)	Holland	4.087
Archives of Gerontology and Geriatrics	13 (3.67)	Ireland	4.163
Social Indicators Research	11 (3.11)	Holland	2.935
Psychology and Aging	9 (2.54)	United States	4.201
Gerontologist	8 (2.26)	United States	5.422
Quality of Life Research	8 (2.26)	Holland	3.44
European Journal of Ageing	6 (1.69)	Germany	3.721

### Category analysis

**FIGURE 2:**
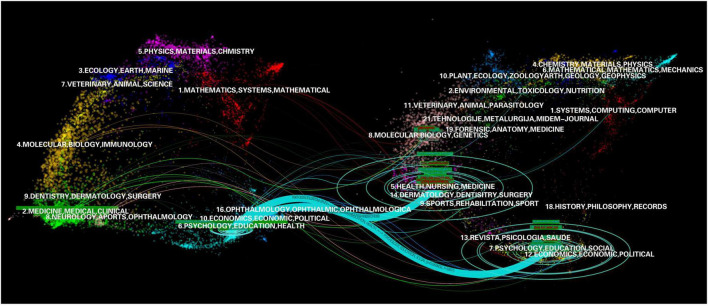
Dual map overlay of journals publishing research on the SWB in older adults. Citing journals are on the left, cited journals are on the right, and the colored paths between them indicate the citation relationship.

Figure 2 shows the dual overlay map of the discipline categories and journals. The dual map overlay of journals as knowledge carriers shows the communication and interaction between disciplines. In the map, the citing part is on the left, and the cited part is on the right, and different lines represent different citation paths. By adjusting the Z-score value, some of the more prominently citing paths can be represented by combined lines, where the larger the circle ripple, the greater the number of journals. As shown in Figure 2, the journals that published articles on the SWB of older adults focus primarily on two disciplines: *psychology, education, and health* and *medicine, medical, and clinical.* In addition, economics, psychiatry, ophthalmology, virology, mathematics, and numerous other disciplines were also involved in varying degrees. There were only two obvious citation paths in the atlas, which originated from *psychology, education, and health*: the first mainly referred to *health, nursing, and medicine*, and the second mainly referred to *psychology, education, and social*. This indicated that the disciplinary basis for research on the SWB of older adults is psychology and medicine.

### Co-institution analysis

**FIGURE 3:**
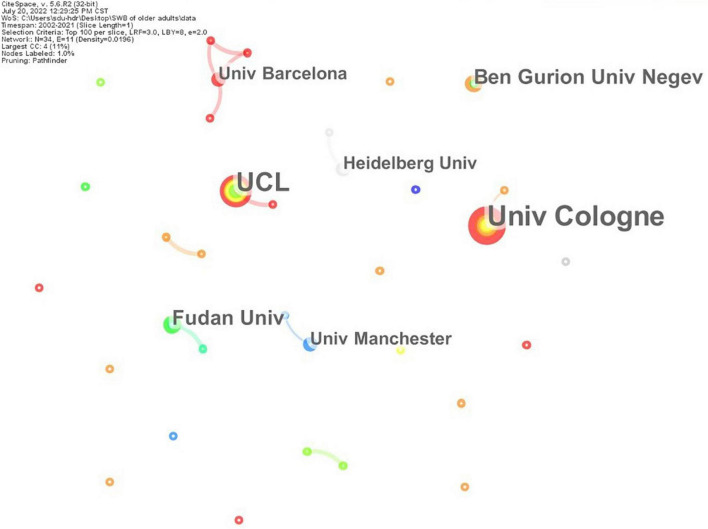
Co-authorship network of organizations. A circle represents an institution, and the size of the circle represents the frequency of publications. The connection between nodes represents the cooperation relationship, and the thickness of the line represents the frequency of collaboration.

Figure 3 shows the cooperative relationship between institutions publishing in the research field of SWB in older adults and institutions with a large number of publications. Table 2 shows the details of publishing institutions. *University of Cologne* (Germany; eight times) was the institution with the most publications, followed by the *University College London* (England; seven times), *Fudan University* (China; four times), and *Ben-Gurion University of the Negev* (Israel; four times). The map showed that the cooperative network was relatively sparse and that there is little cooperation between institutions.

**TABLE 2:** The top seven institutions for publications.

Institution	Frequency	Country	Year
University of Cologne	8	Germany	2019
University College London	7	England	2018
Fudan University	4	China	2016
Ben-Gurion University of the Negev	4	Israel	2017
Heidelberg University	3	Germany	2003
University of Barcelona	3	Spain	2021
University of Manchester	3	England	2014

“Year” indicates the earliest publication time of the institution in 20 years.

### Co-author analysis

**FIGURE 4:**
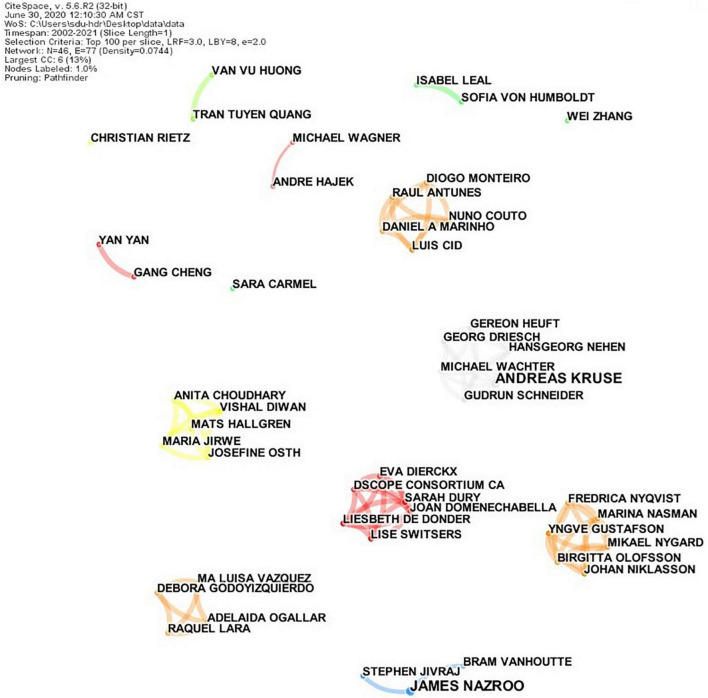
Co-authorship network of authors. A circle represents an author, and the size of the circle represents the frequency of publications. The line between nodes represents the collaboration relationship, and the thickness of the line represents the frequency of collaboration.

Figure 4 shows the co-occurrence network of authors. In the map, apart from Nazroo James and Andreas Kruse, the publications of the two nodes was 3, the other nodes were 2, and there was no high-yield scholar. The network density was 0.0744, which indicated that the overall cooperative network was relatively sparse. Table 3 shows authors with high publication frequency and those with high co-citation frequency in the co-occurrence network. Author co-citation refers to the co-citation relationship formed by two authors being cited by one or more papers simultaneously in this limited field. The author co-citation analysis enabled us to identify scholars with high contributions to the field. Results showed that Ed Diener (201 times, United States) was cited most frequently, followed by Martin Pinquart (76 times, Germany), M. Powell Lawton (72 times, United States), Paul B. Baltes (61 times, Germany), and other authors mainly from the United States, Germany, and the United Kingdom. The World Health Organization (47 times, Switzerland) was the only organization, and all other authors were individuals. A comparison of the two parts of the information in the table indicated that there was no direct relationship between the frequency of posting and the frequency of co-citation. There was no high-publication author in this field, and cooperation was relatively weak. However, Diener E and others have significantly influenced this field.

**TABLE 3:** Top 10 authors for publication frequency and co-citations.

Frequency	Author	Year	Country
**Based on publication frequency**			
3	Nazroo James	2014	England
3	Andreas Kruse	2003	Germany
2	Yngve Gustafson	2020	Sweden
2	Vishal Diwan	2019	India
2	Nuno Couto	2018	Portugal
2	Tran Tuyen Quang	2018	Vietnam
2	Stephen Jivraj	2014	England
2	Sofia Von Humboldt	2017	Portugal
2	Sarah Dury	2021	Belgium
2	Sara Carmel	2017	Israel
**Based on co-citations**			
201	Ed Diener	2003	United States
76	Martin Pinquart	2006	Germany
72	M. Powell Lawton	2003	United States
61	Paul B. Baltes	2005	Germany
47	World Health Organization	2012	Switzerland
45	Carol D. Ryff	2006	United States
41	Andrew Steptoe	2014	England
39	Laura L. Carstensen	2006	United States
34	Linda K. George	2009	United States
32	Ann Bowling	2010	England

In the co-occurrence network of authors, a total of 44 authors published with a frequency of 2, and 8 were randomly selected for presentation. “Year” indicates the earliest publication time or cited time of the author in 20 years.

### Distribution of countries

**FIGURE 5:**
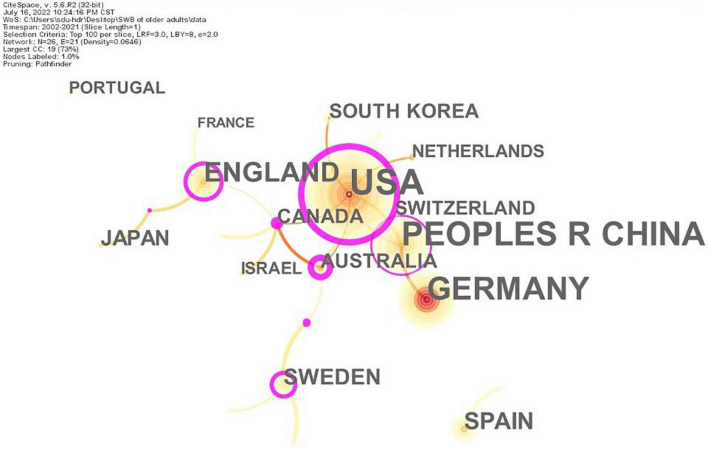
Co-authorship network of countries. A circle represents a country, and the size of the circle represents the frequency of publications. The connection between nodes represents the cooperation relationship, and the thickness of the connection represents the frequency of cooperation. The purple edge of the node indicates high betweenness centrality (≥0.1).

**FIGURE 6:**
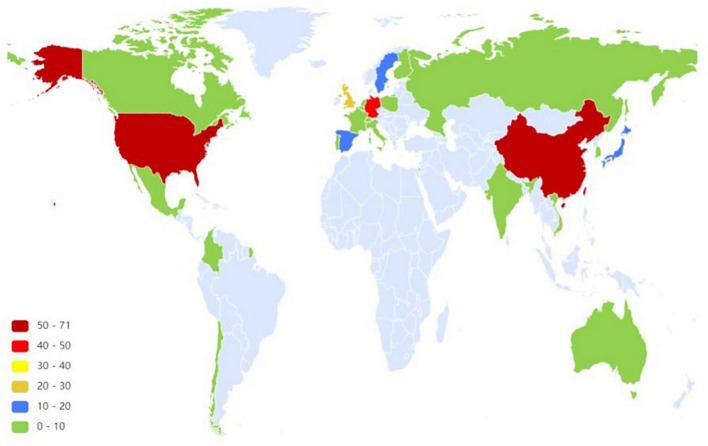
Geographical distribution of publications. The light blue area means that the number of publications is 0.

Figure 5 shows the cooperation between different countries in the research field of the SWB of older adults and countries with numerous publications. Table 4 shows the frequency and betweenness centrality of countries with a large number of publications in the cooperation network. The United States had the highest frequency of occurrence (71) and high betweenness centrality (0.49), which indicated that it had the largest influence in the cooperative network, followed by China and Germany, with 51 and 46 publications, respectively. The high betweenness centrality of China indicated that it also had considerable influence. The United Kingdom, Spain, Sweden, and Japan had fewer publications, and Canada, at number 8, had the highest betweenness centrality of 0.54. Overall, developed countries were the main contributors to the cooperation network. Connections within the cooperative network revolved primarily around the United States, which indicated that the United States held a core position in the cooperative network. China was the largest developing country and played a prominent role in the cooperation network. The geographic visualization provided in Figure 6 showed that the abovementioned countries were mainly concentrated in Europe, North America, Asia, and Oceania.

**TABLE 4:** Top 15 countries for the number of publications.

Country	Frequency	Centrality	Year
United States	71	0.49	2002
China	51	0.11	2010
Germany	46	0.00	2003
England	28	0.31	2013
Spain	15	0.00	2014
Sweden	13	0.22	2019
Japan	11	0.00	2007
Canada	10	0.54	2011
South Korea	10	0.00	2013
Australia	10	0.46	2011
Switzerland	8	0.00	2019
Netherlands	7	0.00	2006
Israel	6	0.00	2017
Portugal	6	0.00	2017
France	4	0.00	2015

“Year” indicates the earliest publication time of the country in 20 years.

### Co-citation analysis of references

**FIGURE 7:**
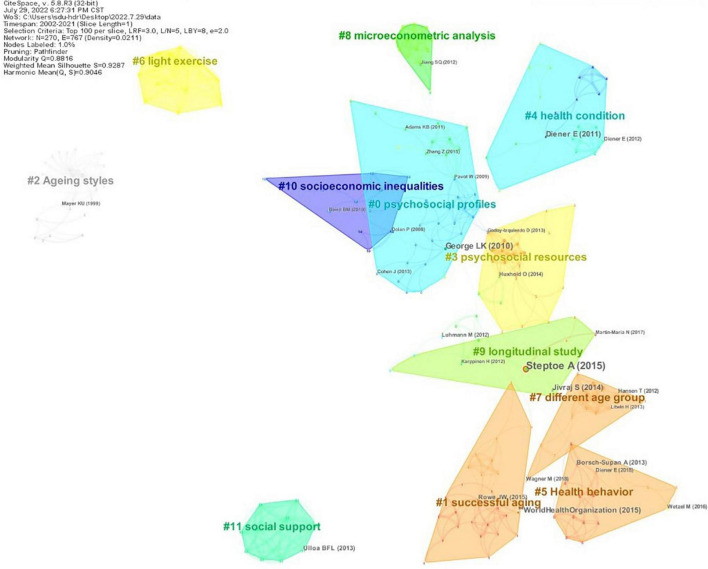
Reference co-citation clusters. Each color block represents a cluster of co-cited references. The number of clusters is sorted according to the size of the color block (i.e, the number of references included in the cluster).

Reference co-citation refers to the frequency of two references being cited by the same reference in a specific research field. The co-citation clustering of references can better identify the research frontier. Reference co-citation clustering divides references into several subfields according to the degree of connection between references. From the perspective of bibliometrics, a highly cited reference in the subfield is considered the knowledge base as the knowledge carrier of the field (Chen et al., 2010; Zhang et al., 2022), whereas the citing reference that cites more papers in this subfield is considered the research front in the field. The citing references shape the clusters, indicating the main research interests and trends of the clusters (Chen et al., 2014a,b). In CiteSpace, the log-likelihood ratio algorithm generated co-citation clusters, and label names were extracted from the titles of citing references, as shown in Figure 7. The Q and S values of the cluster map were 0.8816 and 0.9287, respectively, which indicated that the research results were reasonable and reliable. Table 5 shows the top 12 clusters by size and lists their label name, size, silhouette values, the top three high-frequency publications (and times cited ≥4) of the cited references, as well as information on the top three active citing references (≥5). In addition, if these references met the above requirements and the numbers were equal, they were included together. The internal silhouette values of the generated reference co-citation clusters were generally high and close to 1, indicating good internal consistency. If this value was 1, it indicated that the cluster was independent of other clusters.

**TABLE 5:** Reference co-citation clustering results.

Cluster	Size	Silhouette	Highly cited references	Highly citing references
#0 psychosocial profiles	35	0.955	[14] (George, 2010); [4] (Pavot and Diener, 2009); [4] (Cohen, 2013); [4] (Adams et al., 2011);[4] (Zhang and Zhang, 2015)	[8] (Moreno et al., 2014); [7] (Zhang and Zhang, 2017); [5] (Montizaan and Vendrik, 2014)
#1 successful aging	26	0.931	[11] (World Health Organization [WHO], 2015); [6] (Rowe and Kahn, 2015)	[8] (Plugge, 2021); [7] (Wu et al., 2020); [6] (Tian et al., 2020)
#2 ageing styles	24	1.000	[4] (Staudinger et al., 1998)	[17] (Schneider et al., 2003a); [17] (Schneider et al., 2003b)
#3 psychosocial resources	23	0.925	[5] (Huxhold et al., 2014); [4] (Godoy-Izquierdo et al., 2013)	[12] (Lara et al., 2020b); [10] (Lara et al., 2020a); [8] (Yu et al., 2020);
#4 health condition	20	0.936	[10] (Diener and Chan, 2011); [5] (Diener, 2012)	[7] (Liu et al., 2016b); [6] (Liu et al., 2016a); [5] (Wahl, 2013)
#5 health behavior	17	0.990	[6] (Borsch-Supan et al., 2013); [5] (Wetzel et al., 2016); [5] (Diener et al., 2018)	[6] (Stenlund et al., 2021); [5] (Martín-María et al., 2021)
#6 light exercise	15	1.000	None	[15] (Choudhary et al., 2019)
#7 different age group	15	0.980	[5] (Wagner et al., 2018); [5] (Hansen and Slagsvold, 2012); [4] (Litwin and Stoeckel, 2012)	[11] (Nasman et al., 2020b); [10] (Nasman et al., 2020a)
#8 microecono-metric analysis	13	0.975	[4] (Jiang et al., 2012)	[11] (Tran and Vu, 2018); [11] (Tran et al., 2017)
#9 longitudinal study	13	0.935	[32] (Steptoe et al., 2015); [10] (Jivraj et al., 2014); [5] (Luhmann et al., 2012)	[10] (Cheng et al., 2022); [5] (Carmel et al., 2017)
#10 socioeconomic inequalities	11	0.987	[4] (Dolan et al., 2008); [4] (Baird et al., 2010)	[6] (Jivraj et al., 2014); [6] (Jivraj and Nazroo, 2014)
#11 social support	10	1.000	[7] (López Ulloa et al., 2013)	[10] (Tian, 2014); [9] (Wang, 2014)

Numbers in square brackets indicate citation frequency.

Cluster #0 was the largest cluster named *psychosocial profiles*, with a total of 35 articles. Among these, George (2010) was the most cited paper, which summarized studies on the SWB in older adults published before 2010. The study discussed the concept and definition of SWB in older adults and other fundamental issues and summarized the research progress of the research field. Pavot and Diener (2009) is part of Assessing Well-being, a comprehensive review of the Satisfaction With Life Scales (SWLS). Cohen (2013) is a tool book on the principles of statistics; its content has been widely cited in statistics-related studies. Adams et al. (2011) reviewed studies on the association between the activities of older adults and SWB, focusing on the conceptualization of these activities (e.g., activity classification), measurement methods, and research status related to SWB. Zhang and Zhang (2015) explored the effect of social participation on retirees’ SWB. Results showed that the three aspects of social participation (i.e., frequency of participation in social activities, the role played in social activities, and participation in the activities of the original employer) were positively correlated with SWB, except for work status. Regarding research frontiers, cluster #0 payed more attention to the psychosocial functioning of older adults. Moreno et al. (2014) used a person-centered, multidimensional, and empirically-derived method to conduct a cluster analysis of profiles of psychosocial functioning in older adults, and identified three aging trajectories with different psychosocial characteristics, indicating that SWB depends on different psychosocial configurations. Zhang and Zhang (2017) focused on the relationship between perceived neighborhood support and SWB of the older adults, and the study showed that community awareness played a mediating role. Montizaan and Vendrik (2014) explored the impact of social comparison on job satisfaction among the older adults in the Dutch pension system reform. Research has shown that income has a higher impact on job satisfaction than well-being, and the decline in job satisfaction is strongly influenced by social comparison among colleagues.

Cluster #1, named *successful aging*, contained 26 articles in total and focused on successful aging. The World Report on Aging and Health was the most cited paper, which was published by the World Health Organization in 2015. Based on evidence from different countries, this detailed guidance on health and aging influenced policy-making and opened new avenues for future research. Rowe and Kahn (2015) commented on the MacArthur model of successful aging. Regarding research frontiers, Wu et al. (2020) and Tian et al. (2020) both focused on serious leisure involvement. Both successful aging and active aging theories emphasize the social participation of older adults, and serious leisure involvement is considered an important form of social participation. Finally, Plugge (2021) examined successful aging in people aged over 80 years and found significant differences between subjective and objective criteria for successful aging.

Cluster #2, named *Ageing styles*, contained 24 articles and focused on aging in older adults. The most cited study was by Staudinger et al. (1998), whose research topic was the relationship among older adults’ self, various aspects of personality (e.g., personality characteristics, self-definition, time experience, and others), and aging satisfaction. Regarding research frontiers, two papers by Schneider G were highly citing references. One study showed that subjective physical discomfort of older adult patients who experienced a delay in hospitalization is not determined by objective health status but is related to SWB. Thus, subjective physical discomfort may be used as an evaluation index to evaluate somatization. Another study investigated different aging styles in older adult hospitalized patients and whether there is a difference between self-rated subjective body complaints and those assessed by mental health professionals. The study found a difference, suggesting that perceived physical complaints should be included in the evaluation of SWB.

Cluster #3 was named *psychosocial resources* and contained 23 articles. Huxhold et al. (2014) studied the influence of social activities on the SWB of middle-aged and older adults, and showed that social activities affect negative aspects and life satisfaction in older adults but not middle-aged adults. Whereas Godoy-Izquierdo et al. (2013) explored current and past happiness levels of older adults. Regarding research frontiers, cluster #2 focused on the psychosocial resources of older adults. Two articles by Lara et al. were highly citing references and explored the relationship between various social-psychological resources and SWB. They found that self-efficacy significantly influences SWB but does not directly affect SWB; rather, it is mediated entirely by perceived health. Self-efficacy has also been shown to play a fundamental role in various social-psychological resources. Yu et al. (2020) showed that two psychological needs, relatedness and competitiveness, are significantly associated with SWB of the older adults, but autonomy is not.

Cluster #4, named *health condition*, contained 20 references and focused on the health condition of older adults. The knowledge base of this subfield comprised two reviews on SWB by Diener E. Diener and Chan (2011) reviewed seven different studies on the relationship among SWB, health, and longevity, and showed that high SWB makes the latter two better. Diener (2012) described the recent findings on SWB and future research priorities, and importantly, it also found that high SWB benefits health and longevity. Regarding research frontiers, two articles by Liu et al. explored the influence of body mass index (BMI) and visual impairment, respectively, on the SWB of older adults aged over 95 years. The former suggested that low BMI in long-lived women is an indicator of poor psychological well-being, and the latter showed that visual impairment in long-lived individuals (LLIs) was independently associated with the SWB. Another article Wahl (2013) was a summary review of the psychological consequences of visual impairment in later life, including consequences related to SWB.

Cluster #5 was named *Health behavior* and contained 17 references. Borsch-Supan et al. (2013) was the most cited and systematically introduced data from the Survey of Health, Ageing and Retirement in Europe (SHARE). Wetzel et al. (2016) showed that retirement has short-term and long-term effects on life satisfaction in older adults, depending on the last labor market status and educational status, respectively. Diener et al. (2018) reviewed the research progress of SWB and introduced the main research fields at the time. Regarding research frontiers, cluster #5 mainly focused on the health behavior of older adults. Stenlund et al. (2021) confirmed the predictive effect of the health behavior total score composed of eating habits, smoking, drinking and physical activity on the life satisfaction score, indicating that a good behavior and lifestyle can effectively promote life satisfaction. Martín-María et al. (2021) explored the longitudinal effects of health behaviors on different dimensions of SWB, while investigating gender differences, and finally showed differences in the relationship between the frequency of healthy behaviors and SWB levels in men and women.

Cluster #6, named *light exercise*, contained 15 references and mainly focused on yoga interventions. All references in the cluster were co-cited twice, and the silhouette value was 1, which indicated that the cluster had no connection with any of the other clusters. This suggested that there is no outstanding research foundation in this subfield. Regarding research frontiers, there were only two highly citing references in this subfield, both of them were study protocols, and the research topic was the influence of yoga interventions on the SWB of older adults.

Cluster #7, named *different age group*, contained 15 references and highly focused on age differences in SWB. Wagner et al. (2018) briefly reviewed the challenges and potentials model of quality of life of the very old and its existing research, focusing on an overview of “Quality of life and subjective well-being of the very old in North Rhine-Westphalia” (NRW80 +) project. Hansen and Slagsvold (2012) explored the age difference in the major outcomes of SWB (e.g., life satisfaction, positive affect and negative affect), and found that the changes of each dimension of SWB were not uniform, and SWB was stable or increased only in young-old age, while SWB declined in later years. Litwin and Stoeckel (2012) focused on age differences in the influence of social networks on SWB, and showed that social networks have different effects on the very old than on the young-old person. Regarding research frontiers, two papers by Nasman M were citing references, Nasman et al. (2020b) explored the differences in the morale of the older adults in different age groups, and found that older age was independently associated with lower morale. Nasman et al. (2020a) focused on morale in very old people, and found that the development of depression and increased loneliness were significant risk factors for lower morale.

Cluster #8, named *microeconometric analysis*, contained 13 references and paid more attention to microeconometric analysis. Jiang et al. (2012) used survey data from the 2002 Chinese Household Income Project (CHIP) to find that inequality was positively associated with well-being when controlling for identity-related inequality and other individuals, family, and city-level characteristics. Regarding research frontiers, two references by Tran TQ were highly citing references and explored the influence of housing and economic inequality, respectively, on the SWB of older adults using the national aging data of Vietnam. The former found that housing satisfaction in older adults was predictive of life satisfaction, while the latter showed that economic inequality negatively affects the quality of life of older adults.

Cluster #9, named *longitudinal study*, contained 13 references and mainly focused on how the SWB of older adults changes with age. Steptoe et al. (2015), which was published in The Lancet, proposed a new analytical idea for the happiness patterns of different age groups and reported a correlation between happiness and mortality. The study found that existing psychological or economic theories do not fully explain the happiness patterns that change with age. Moreover, they found a significant correlation between happiness and survival rate. However, a causal relationship was not established because potential confounding factors could not be ruled out. Jivraj et al. (2014) explored various longitudinal relationships among SWB, cohort age, and aging, and found that cohort differences were not the cause of low well-being. Adams et al. (2011) reviewed studies on the association between the activities of older adults and SWB, focusing on the conceptualization of these activities (e.g., activity classification), measurement methods, and research status related to SWB. Zhang and Zhang (2015) explored the effect of social participation on retirees’ SWB. Results showed that the three aspects of social participation (i.e., frequency of participation in social activities, the role played in social activities, and participation in the activities of the original employer) were positively correlated with SWB, except for work status. Luhmann et al. (2012) conducted a longitudinal meta-analysis of the different effects of life events on the two components of SWB (affective well-being and cognitive well-being, respectively), indicating that life events have a more substantial effect on cognitive well-being. Regarding research frontiers, Cheng et al. (2022) and Carmel et al. (2017) both conducted longitudinal studies. The former found gender and residence differences in the impact of social support on the SWB of older adults, and the latter found that personal resources and appropriate coping behaviors positively affected the SWB of older adults.

Cluster #10, named *socioeconomic inequalities*, contained 11 articles and mainly focused on economic research perspective. Dolan et al. (2008) was the most cited paper, and it reviewed the research on subjective well-being and its influencing factors in mainstream economics journals. The research showed that personal, social and background factors that do not change over time can effectively predict well-being. Baird et al. (2010) explored the relationship between life satisfaction and age using data from two national surveys: the German Socio-economic Panel Study and the British Household Panel Study. The study found that age-related life satisfaction followed a U-shaped curve, although the results of the two data studies differed. Regarding research frontiers, Jivraj et al. (2014) was a highly citing reference and also highly cited reference in cluster #8. Jivraj and Nazroo (2014) pointed out that health, social, and economic inequalities are objective factors that contribute to differences in the SWB of older adults in their later years.

Cluster #11, named *social support*, contained 10 articles and focused on social support for older adults. López Ulloa et al. (2013) summarized the theoretical and empirical studies on the relationship between aging and SWB and showed that there remains considerable controversy on this topic. Regarding research frontiers, Wang (2014) focused on the influence of the social network scale on the SWB of older adults, and confirmed the mediating role of perceived social support. Tian (2014) investigated the influence of intergenerational social support on the SWB of older adults, and showed that self-esteem and loneliness played a mediating role.

In summary, the knowledge base of each subfield of SWB research mostly comprises basic theoretical research, which was predominantly presented in the form of reviews. Diener E has laid a theoretical foundation for the field of the SWB of older adults as well as the SWB field as a whole. In the discussion section, the research fronts of the sub-fields will be sorted out, and the critical sub-topics of the whole field will be reported.

### Keyword analysis

#### Co-occurrence of keywords

**FIGURE 8:**
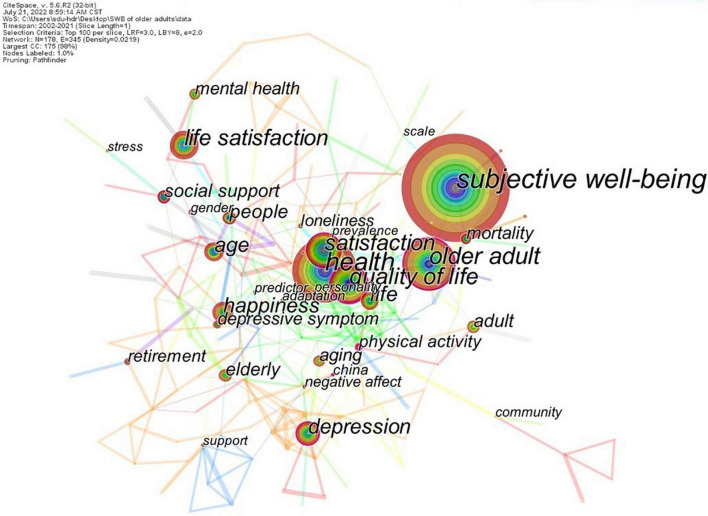
Co-occurrence network of keywords. A circle represents a keyword, and the size of the circle represents the frequency of occurrence. The connection between nodes represents the cooperation relationship, and the thickness of the connection represents the frequency of cooperation. The purple edge of the node indicates high betweenness centrality (≥0.1).

Keywords are defined as the most concise set of words in a reference topic. Analysis of keywords captures the core feature of the article. The principle behind keyword co-occurrence is the calculation of the number of times a pair of keywords appear together in the same paper. This can be used to identify research hotspots within a certain field. We merged keywords with the same or similar meanings. For example, we merged “elderly people” with “older adults.” Figure 8 shows the cooperative network for keyword co-occurrence, consisting of 178 nodes and 345 connections. In this figure, *subjective well-being*, *older adult*, *people*, *elderly*, and *adult* were used as search terms in this study. Apart from these keywords, *health* was the largest and had strong links with other keywords. Table 6 shows the frequencies, betweenness centralities, and the earliest year of occurrence for the top 20 keywords. Fifteen keywords in the table had high betweenness centrality, whereas the remaining five had a high frequency of occurrence. The keywords in Table 6 were the most influential in the cooperative network. Of all these keywords, *health*, *depression*, *age*, *social support*, *mental health*, *aging*, and *retirement* were important influencing factors of SWB, and *life satisfaction* is one of the three dimensions for evaluating SWB, and *physical activity* and others were common interventions for improving SWB.

**TABLE 6:** Top 20 high-frequency keywords.

Keywords	Frequency	Centrality	Year
Subjective well-being	200	0.06	2006
Health	126	0.01	2003
Older adult	106	0.14	2006
Quality of life	82	0.24	2007
Satisfaction	76	0.11	2006
Life satisfaction	70	0.07	2002
Depression	53	0.13	2006
Happiness	49	0.04	2011
Life	49	0.10	2006
Age	47	0.10	2009
People	36	0.15	2008
Social support	33	0.10	2009
Adult	33	0.03	2005
Elderly	30	0.06	2011
Mental health	28	0.03	2012
Aging	26	0.07	2017
Depressive symptom	25	0.10	2009
Mortality	24	0.10	2006
Physical activity	22	0.31	2008
Retirement	21	0.03	2011

**“Year” indicates the earliest occurrence of the keyword in 20 years.**

#### Keyword burst

**FIGURE 9:**
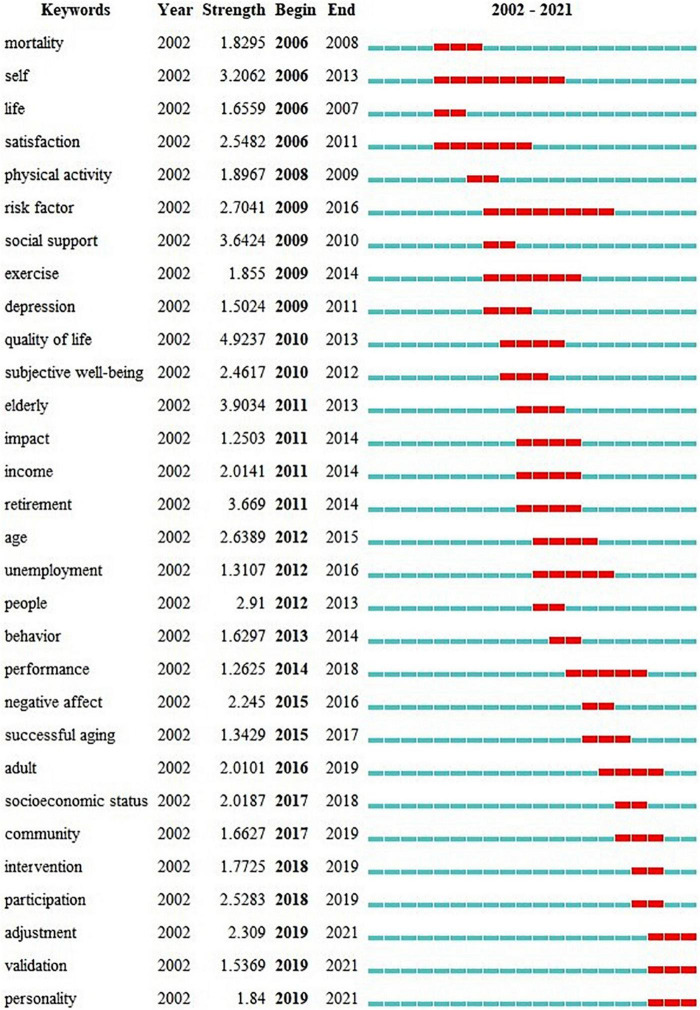
Top 30 keywords with the strongest citation bursts. Red bars indicate the citation bursts of keywords. The strength of a keyword indicates how often it was cited during the burst phase.

Figure 9 shows the top 30 burst keywords, and the time of their burst is marked in red. Keyword citation bursts refer to keywords that have been cited significantly over a period of time, lasting a year or even years (Chen, 2017). Burst keywords are used to identify the research frontier during this period (Wu and Wei, 2022). Analyzing keywords burst map enabled us to explore the evolution of this research field. Figure 9 shows the top 30 burst keywords. As shown in the figure, there were no burst keywords in 2005 or earlier, which may be related to the low publication volume during this period. There were 15 keywords began to burst in the first decade and 15 keywords in the second decade; three of these continued to burst until 2021.

## Discussion

### General information

According to the basic information of the papers, studies on the SWB of older adults have followed a trend of rapid growth over the past 20 years, from 5 studies in 2002 to 53 studies in 2020. In fact, because the source channels of samples in this study were not comprehensive, the actual growth rate may even be faster. In addition, we observed a small peak in 2020, which may be related to the increased focus on the health of older adults due to the COVID-19 outbreak. The 354 articles included in this study were published in 183 journals, covering various disciplines, including gerontology, psychology, medicine, sociology, public health, and numerous other disciplines. The dual overlay map showed that the knowledge base of this field came primarily from psychology and medicine. We also found that the SWB of older adults is gaining increased attention. With the continued development of the field, the disciplines involved will likely become increasingly extensive.

From the perspective of publishing power, the overall cooperative relationship between institutions and authors requires further strengthening. Developed European countries and the United States dominate the research field, whereas East Asian countries of China, Japan, and South Korea, as well as the Oceanic country of Australia, exert some influence in this field. On the one hand, this is related to the high level of economic development in these countries. A good national economic foundation has enabled citizens to increasingly pursue a high level of life quality; in turn, a high level of economic strength and scientific research has allowed more attention to be paid to the SWB of older adults. On the other hand, most of these countries are located in Europe, North America, Asia, and Oceania, where the aging population is relatively prominent. Of these, societies are rapidly aging in Europe, while other societies in North America, Asia, and Oceania are moderately and mildly aging. Therefore, the mental health of older adults is highly valued in these countries.

### Research topic analysis

The reference co-citation cluster analysis, the keyword co-occurrence analysis, and the reference review revealed that the research field of the SWB of older adults can be divided into eight prominent themes.

1. The application of social psychology in the study of the SWB of older adults (cluster #0 *psychosocial profiles* and cluster #3 *psychosocial resources*). As the dual overlay map showed, social psychology is an essential disciplinary basis for the study of SWB. Many studies have shown that psychosocial resources are related to the components of SWB (i.e., affect or hedonic balance and life satisfaction; Blanco-Molina et al., 2019), including self-efficacy, social support, etc. In addition, the study results showed that cluster #0 focused on the social functioning of the psychology of older adults, overlapping with the research content on social well-being, while cluster #3 mainly focused on the psychological resources of the older adults, intersecting with the research content on psychological well-being. Both clusters, although derived from social psychology, have different focuses. It can be seen that the application of social psychology in well-being is the overlapping research content of subjective well-being, psychological well-being, and social well-being, but there is a certain fragmentation state. Further integration of the application of social psychology in well-being may facilitate the fusion of the three well-being research orientations.
2. Aging in older adults (cluster #2 *Ageing styles*). *Aging* (*with a frequency of 26 and a betweenness centrality of 0.07*) has a high frequency in the keyword co-occurrence network and is a prominent physiological problem faced by the older adults. Therefore, the aging of older adults is also a noteworthy topic. Some cohort studies suggested that aging in older adults is associated with worse SWB (Jivraj et al., 2014). In addition, some studies have shown that in very old age, dysfunction and burden near death can lead to an accelerated decline in well-being (Gerstorf et al., 2008, 2010, 2018).
3. Health condition of older adults (cluster #4 *health condition*). *Health* (*126, 0.01*) was the most influential keyword in the co-occurrence network, followed by s*ubjective well-being*. Meanwhile, health status was one of the most decisive factors for SWB and encompassed a wide range of contents, such as physical health, self-rated health, physical function, and *mental health* (*28, 0.03*). Among these, physical and mental health were two major research hotspots. Early studies focused mainly on physical health, whereas increasing attention is being paid to the influence of mental health on SWB because *depression* (*53, 0.13*), anxiety, loneliness and other adverse psychological states are serious threats to the SWB of older adults.
4. Achieving successful aging (cluster #1 *successful aging* and cluster #5 *Health behavior*). There was a relatively tight association between cluster #5 and cluster #1, as shown by the literature co-citation clustering. Furthermore, as the label of cluster #5, health behavior is also one of the important elements highlighted by successful aging. Therefore, the two clusters are grouped into one topic. Successful aging is a complex system of theory, a relatively ideal state people want to achieve. The measurement of successful aging was mostly based on objective criteria in the past, but now more and more attention is paid to subjective criteria (Rowe and Kahn, 2015), which has caused a collision with SWB research. Some scholars believe that SWB directly impacts successful aging (Strawbridge et al., 2002; Westerhof, 2003), and some scholars believe that SWB can be used as a measure of successful aging (Freund and Riediger, 2003). The relationship between SWB and successful aging still deserves further in-depth exploration.
5. Interventions for SWB (cluster #6 *light exercise*). Developing intervention measures or strategies for the SWB of older adults is a major objective of SWB research. Although research has been ongoing for many years, evidence remains more limited than other topics. Fortunately, there has recently been a small rise in relevant research. After sorting through the retrieved literature, we found that interventions for improving the SWB of older adults are focused on two categories: positive psychological and physical behavior interventions. Positive psychological interventions focus on positive psychology, which aims to cultivate skills and develop positive psychological emotions. Common methods are based on gratitude, forgiveness, and mindfulness (Ramirez et al., 2014). Physical behavior interventions focus on the guidance of behavior. The most common intervention is *physical activity* (*22, 0.31*), which was also a high-frequency and high betweenness centrality keyword. Light exercise, such as yoga, is a form of physical activity, as indicated by the label of cluster #6. However, although scholars have made some attempts, there is still no review to summarize the effects of these interventions on SWB in older adults, and it has not yet been determined which are the most effective and durable.
6. Age differences in SWB research (cluster #7 *different age group* and cluster #9 *longitudinal study*). *Age* (*47,0.1*) also has a high frequency of occurrence and high betweenness centrality in the keyword co-occurrence network. Age is a factor along the time dimension, and numerous studies have shown that health deteriorates with age (Kwak, 2018; Finkel et al., 2020); thus, it is reasonable to speculate that SWB will also deteriorate with age. However, several longitudinal studies conducted in Europe and the United States have indicated that SWB increases with age in the later years (from approximately age 50; Stone et al., 2017). Age differences have aroused a wide range of research interests, and a prominent research hotspot is the U-shaped curve of SWB (Stone et al., 2020).
7. An economic perspective of SWB research (cluster #8 *microeconometric analysis* and cluster #10 *socioeconomic inequalities*). Economy is an important factor that affects the SWB of older adults. It encompasses income, pension, and pension security, etc. The influence of income on SWB has been studied extensively. Research has demonstrated that although absolute income does not significantly affect SWB, relative income after social comparison does significantly impact SWB (Alderson and Katz-Gerro, 2016). Thus, the influence of income on SWB is relative, which was confirmed by the study by Zhu et al. (2020) among older adults in China. However, studying income alone may provide an incomplete view of the mechanism underlying the influence of income on well-being because the relationship between material welfare, such as income, and SWB is complex. Several studies have examined pensions. One study conducted in a developing country found that an increase in the actual value of pensions was the cause of improved SWB in older adults (Kollamparambil and Etinzock, 2019). This was inconsistent with the findings of studies conducted in developed countries, which may be because the SWB of older adults in developed countries is primarily related to non-material factors. Taken together, these studies suggest that the influence of pension policy on SWB is impacted by broader national economic and political background factors.
8. Social support for older adults (cluster #11 *social support*). *Social support* (*33,0.1*) also greatly influences the keyword co-occurrence network, which was extracted as the label of cluster #11. The association between social support and SWB has become a popular research topic, and extensive research has confirmed that improving social support effectively enhances the SWB of older adults (Guedea et al., 2006; Deng et al., 2010). However, social support is a comprehensive concept that includes various types of support, such as support from the spouse, children, generation, friends, other family members, and the community. Research has shown differences among different sources of social support (Merz and Huxhold, 2010), and the complex relationship between these sources requires further investigation. In addition, perceived social support may be more important for the SWB of older adults.

In conclusion, the research on the influencing factors of SWB is one of the most important research directions, and the factors affecting the SWB of older adults at the individual, family and social levels have been widely explored. Second, research perspectives of the factors influencing SWB have developed from simple correlations to complex interactions. In addition to studying SWB as an outcome, several studies have examined this association from the perspective of SWB as an influencing factor, this will be an interesting and necessary direction. Finally, it is worth mentioning that methodological research on measuring SWB is not very prominent, but also lays a solid foundation for this field of research.

### Research trends analysis

As shown in the burst keyword map, *risk factor* and *self* had the longest burst times, which suggested that, among the influencing factors of SWB, greater attention was paid to risk factors. Moreover, more emphasis has been placed on evaluating SWB from the perspective of personal subjectivity. In addition, *quality of life* is the subject of SWB assessments, so it is reasonable to see it as the keyword with the highest burst intensity. An interesting point is that citation keywords like “elderly” burst several years ago because most journals no longer used them. In recent years (i.e., 2017–2021), *socioeconomic status*, *community*, *intervention*, *participation*, *adjustment*, *validation* and *personality* have become current research hotspots. With the continuous advancement of topics (1) and (7), in recent years, the *socioeconomic status* of the older adults and the *community* they live in have attracted more and more attention. As mentioned above, intervention research on SWB has increased in recent years and is likely to also become a research hotspot in the future. Social participation is one of the three pillars of active aging. Older adults can engage in social participation via re-employment, delayed retirement, and voluntary service. *Participation* had the highest intensity among all burst keywords in recent years, which indicated that this aspect has attracted widespread attention. *personality* is closely related to SWB, and research showed that its five traits (neuroticism, extraversion, agreeableness, conscientiousness, and openness to experience) are strong predictors of SWB (Kahlbaugh and Huffman, 2017; Anglim et al., 2020). It is of great significance to further explore the mechanism between them and SWB. Aging is a serious threat to the mental health of older adults, so *adjustment* or adaptation to age-related problems is also an important research topic. In addition, the *validation* of mental health scale validity has also attracted significant attention. Notably, *adjustment*, *validation* and *personality* were bursting through 2021, suggesting that their popularity was more likely to remain in the next few years.

## Conclusion

In this paper, we used CiteSpace to visually analyze 354 papers on the SWB of older adults and examine the current state of the literature. We found that the primary research source was concentrated in regions with prominent aging population and high economic strength, although the cooperation between these regions was generally relatively weak. The reference co-citation clustering analysis revealed that there were eight important topics in this field. The burst keyword map indicated that *socioeconomic status*, *community*, *intervention*, *participation*, *adjustment*, *validation* and *personality* were recent research frontiers.

There remain several areas that require further exploration in this field. First, research findings of various influencing factors may vary because of differences in sample size, research methods, data type, and cultural background. Furthermore, well-being is the result of a combination of factors; thus, results may be affected by unknown factors. Therefore, the results should be interpreted critically, and for controversial findings, reasons for discrepancies must be further examined. Moreover, future research should be optimized to understand how to effectively control for confounding factors when determining the effect of influencing factors. Secondly, these influencing factors are not always independent, and many are interrelated. In addition, the potential reverse effects of SWB on these influencing factors and broader health outcomes have not been sufficiently explored, and the mechanisms underlying the effect of the influencing factors require further study. Finally, SWB research is gradually entering the deep water area, and intervention studies for improving SWB have gained wider attention. Thus, to facilitate the development of new interventions, it is necessary to clarify the association between influential factors via meta-analyses (Diener et al., 2018).

This study has several limitations. Firstly, we only used the Web of Science Core Collection as the retrieval channel, and our retrieval formula did not include “happiness” or other synonyms of SWB. Therefore, the literature search may not have been sufficiently comprehensive, which may have introduced bias. Secondly, because of the scarcity of relevant studies in low and middle income countries, some of our conclusions may not apply to some countries. Finally, CiteSpace has several limitations, such as the inability to accurately distinguish the first author, corresponding author, or other authors. Nevertheless, in general, the study was based on objective data, which minimized bias.

## Author contributions

DH conceived and designed the manuscript, ran the software, analyzed and interpreted the results, and wrote the first draft of the manuscript. JW proofread the final draft and controlled the quality of the articles. HF, YZ, and SC collected and organized the literature and revised the manuscript. XW proofread the manuscript and processed the figures and tables. All authors contributed to the article and approved the submitted version.
